# Precautions When Extracting Impacted Third Molars With Impacted Enamel Pearls in the Maxillary Sinus Floor: A Case Report

**DOI:** 10.1002/ccr3.73400

**Published:** 2026-08-26

**Authors:** Hideki Suito, Yuuri Oku, Tomoyuki Kondo, Kumiko Kamada, Naito Kurio, Naoki Maeda

**Affiliations:** ^1^ Department of Oral and Maxillofacial Radiology, Graduate School of Biomedical Sciences Tokushima University Tokushima Japan; ^2^ Department of Oral Surgery, Graduate School of Biomedical Sciences Tokushima University Tokushima Japan

**Keywords:** cone‐beam computed tomography, enamel pearl, maxillary sinus floor, multidetector computed tomography

## Abstract

Cone‐beam computed tomography (CBCT) is routinely used before mandibular wisdom tooth extraction to determine its three‐dimensional relationship with the mandibular canal. In contrast, the extraction of a maxillary third molar is often planned based on panoramic radiographs alone. Enamel pearls are common at the maxillary molar sites, with prevalence varying by ethnicity; however, their detection based on panoramic radiographs alone is challenging. We herein present the novel case of a 27‐year‐old woman whose maxillary sinus floor was being elevated and partially perforated by enamel pearls, thereby increasing the risk of sinus perforation area spread, enamel pearl fracture, and displacement into the sinus cavity during extraction as well as postoperative complications. This case illustrates the clinical implications for the surgical management of impacted maxillary third molars located close to the maxillary sinus floor and also emphasizes the value of CBCT imaging for accurate preoperative assessment and surgical planning in such cases.

## Key Clinical Message

Enamel pearls may elevate or perforate the maxillary sinus floor when associated with impacted third molars. Unlike panoramic radiographs, cone‐beam computed tomography enables precise detection of enamel pearls and their relation to the sinus, supporting safe surgical planning and preventing postoperative complications such as perforation or pearl retention.

## 1 Introduction

Enamel pearls are formed from Hertwig's epithelial sheath. When dentin begins to form in the tooth root, the epithelial sheath normally collapses and detaches from the surface of the tooth root, prompting the contact of the dental follicle cells with the predentin and their differentiation into cementoblasts, initiating the formation of cementum. However, epithelial sheath cells that attach to and remain on the predentin differentiate into fully functional ameloblasts, which produce enamel, leading to the formation of enamel pearls [1, 2].

Enamel pearls are present in approximately 1%–9% of the population, with prevalence varying by ethnicity and diagnostic/examination methods. Many reports on enamel pearls have focused on the examination of periodontal diseases caused by enamel pearls [3, 4, 5], enamel pearl structure [6, 7], size [6, 7, 8, 9], and differences in prevalence by race [9, 10] and by race and tooth type [11, 12, 13]. These and other reports have indicated that enamel pearls are more frequent in molars, particularly maxillary molars, than in anterior teeth or premolars [14, 15]. Regarding their location in the maxillary molars, enamel pearls are most common on the distal root surface [16]. To the best of our knowledge, we are unaware of previous reports that have evaluated maxillary sinus floor elevation by enamel pearls in an impacted maxillary third molar.

## 2 Case History/Examination

**FIGURE 1:**
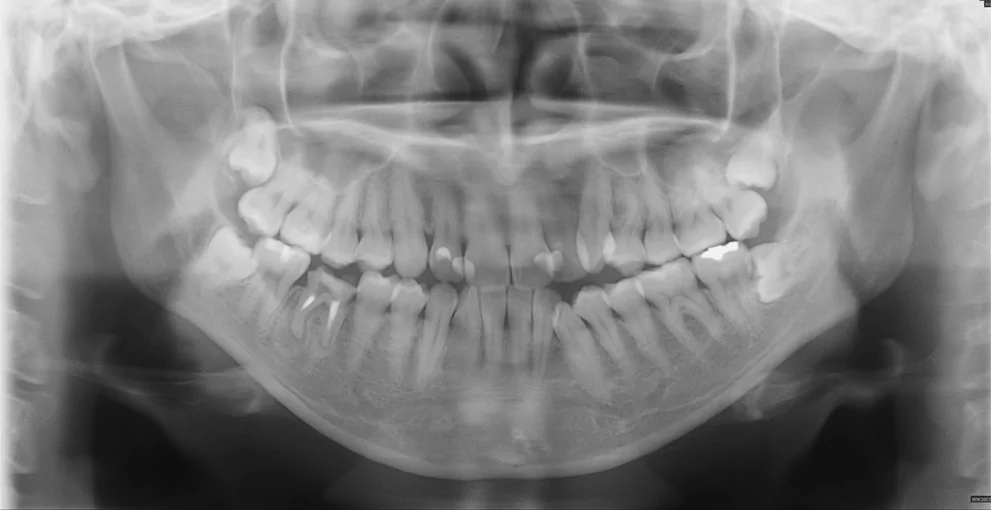
A panoramic radiograph image. Panoramic radiographs taken at the initial visit on December 11, 2023. Enamel pearls cannot be confirmed at the maxillary left third molar site in the left maxillary sinus floor.

A 27‐year‐old woman, with a referral from another orthodontic clinic, visited the oral surgery department of our clinic for surgical orthodontic treatment on December 11, 2023. Owing to mandibular protrusion and maxillary deficiency, we planned LeFort I osteotomy for the maxillary bone as well as sagittal split ramus osteotomy. Panoramic radiographs (Figure 1) were taken on the same day to aid in the extraction of maxillary and mandibular third molars. In addition, for postoperative simulation, we performed imaging with multidetector computed tomography (MDCT; Aquilion ONE/ViSION Edition [320 rows; Canon Medical Systems Co., Tochigi, Japan]) on January 5, 2024. The imaging was conducted with helical scanning under the following conditions: a tube voltage of 120 kV, a tube current of 300 mA, a field of view (FOV) of 160 mm, and a rotation time of 0.5 s/rot. The reconstruction was performed with a slice thickness of 0.5 mm and a reconstruction interval of 0.5 mm using Adaptive Iterative Dose Reduction 3D (AIDR 3D), which is a successive approximation image reconstruction method.

**FIGURE 2:**
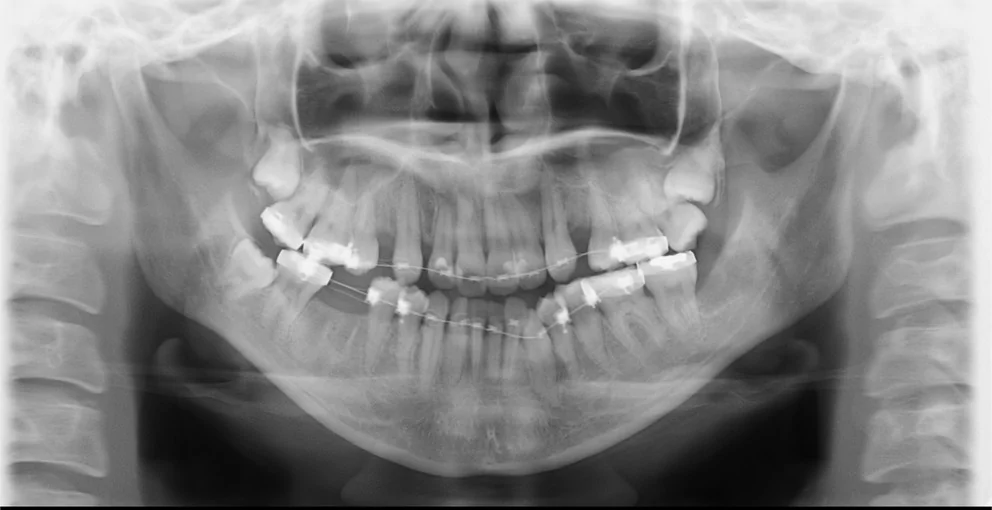
A panoramic radiograph image. The patient underwent extraction of teeth the mandibular right first molar, the mandibular left third molar and the maxillary right first premolar, the maxillary left first premolar at another dental practice and returned to our clinic for extraction of teeth the mandibular right third molar, the maxillary right third molar, the maxillary left third molar. Panoramic X‐ray taken during this visit on July 5, 2024. Again, enamel pearls cannot be confirmed at the maxillary left third molar site in the left maxillary sinus floor.

Subsequently, the patient underwent the extraction of the mandibular right first molar, the mandibular left third molar and the maxillary right first premolar, the maxillary left first premolar at another clinic, and she revisited our clinic for the extraction of the mandibular right third molar, the maxillary right third molar, the maxillary left third molar. The panoramic radiographs taken at this visit (Figure 2) showed the curved root of the maxillary left second molar owing to the maxillary left third molar crown. Based on this finding, we proposed the following treatment plan: (1) the maxillary left third molar would be extracted if the root condition of the maxillary left second molar was favorable, or (2) after the extraction of the maxillary left second molar, the maxillary left third molar would be retracted and allowed to erupt if the root condition of the maxillary left third molar was favorable. Thus, we performed CBCT imaging to examine the root conditions of the maxillary left second molar and the maxillary left third molar and their relationship with surrounding tissues on September 5, 2024. CBCT imaging was performed using 3D Accuitimo (J. MORITA Corp, Osaka, Japan) under the usual imaging conditions used at our clinic (a tube voltage of 80 kV, a tube current of 5.0 mA, an FOV of Φ60 × H60 mm, a voxel size of 125 μm, a reconstruction slice thickness of 0.5 mm, and a slice interval of 0.5 mm). The image viewing software used was I‐Dixel (J. MORITA Corp).

## 3 Differential Diagnosis, Investigations and Treatment

**FIGURE 3:**
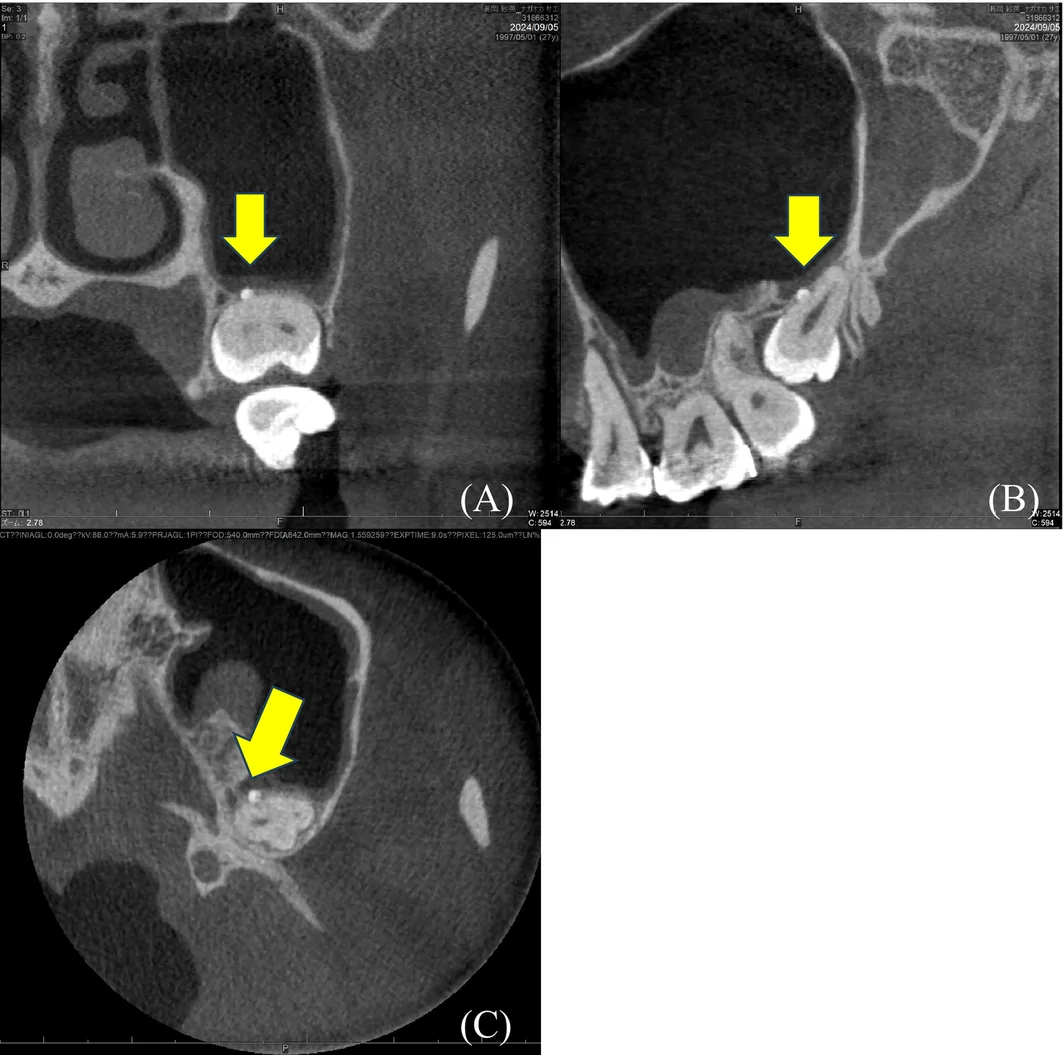
Cone‐beam computed tomography images. Image acquired on September 5, 2024, using 3D Accuitimo (J. MORITA Corp, Osaka, Japan) under the usual imaging conditions used at our clinic (a tube voltage of 80 kV, a tube current of 5.0 mA, an FOV of Φ60 × H60 mm). (A) Coronal slice image, (B) Sagittal slice image, (C) Horizontal slice image. A semicircular, highly absorbent image of an enamel pearl was observed in the area indicated by the arrow.

**FIGURE 4:**
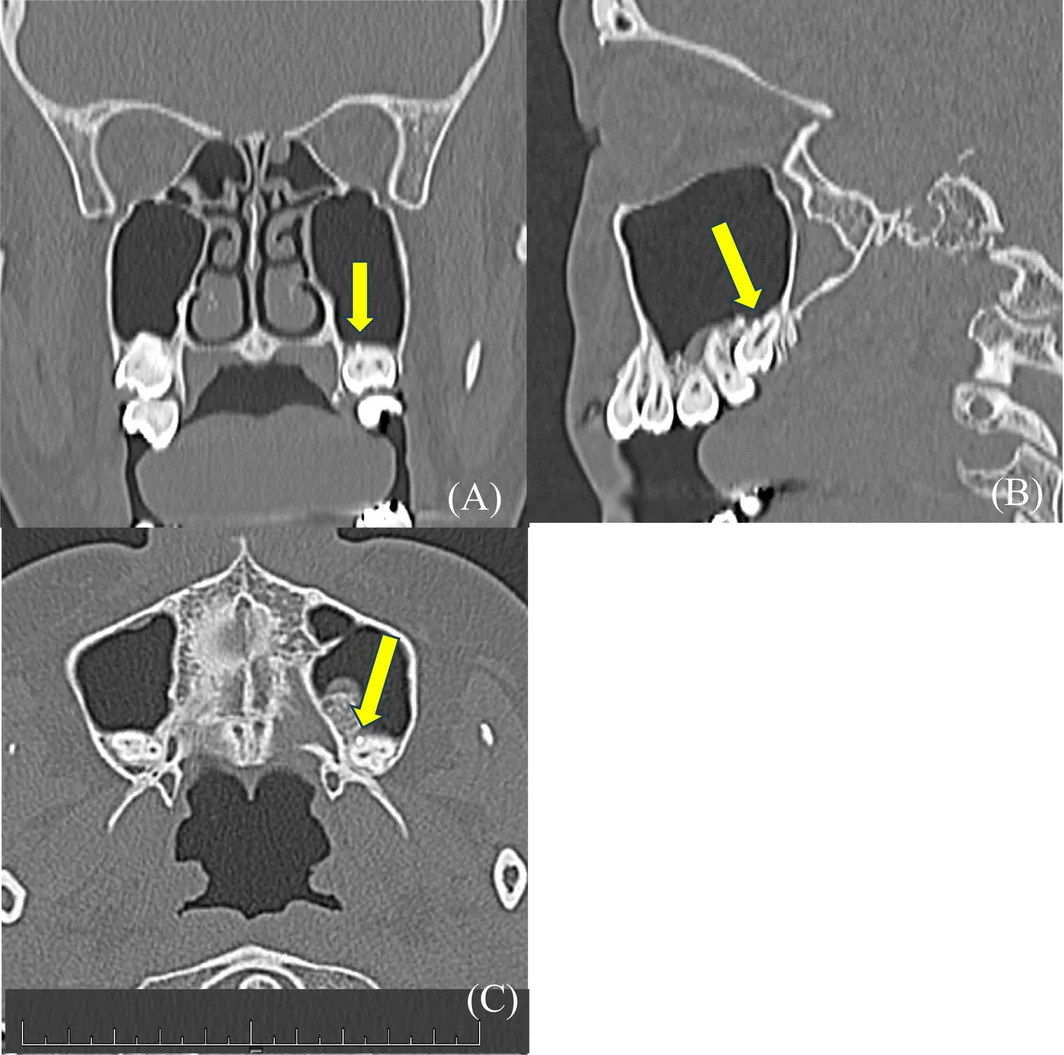
Multidetector computed tomography images. Images acquired on January 5, 2024, using multislice computed tomography (MDCT; Aquilion ONE/ViSION Edition (320‐slice; Canon Medical Systems Corporation, Tochigi Prefecture)) for postoperative simulation. (A) Coronal slice image, (B) Sagittal slice image, (C) Horizontal slice image. A semicircular, highly absorbent image of an enamel pearl was observed in the area indicated by the arrow.

CBCT images indicated that the enamel pearls were 1.4 mm in major axis, 1.3 mm in minor axis, and 1.0 mm in height. The images showed an elevated maxillary sinus floor owing to enamel pearls and indicated a loss of continuity in part of the maxillary sinus floor (Figure 3). In addition, the MDCT images taken on January 5, 2024, suggested the presence of enamel‐like structures based on the difference in the Hounsfield unit (HU) value compared with that of the surrounding area (Figure 4). However, the MDCT images did not sufficiently indicate the morphology and size of the enamel pearls or the presence (or absence) of perforation into the maxillary sinus despite the mean CT value of 1827 HU, which was highly comparable to that of enamel.

**FIGURE 5:**
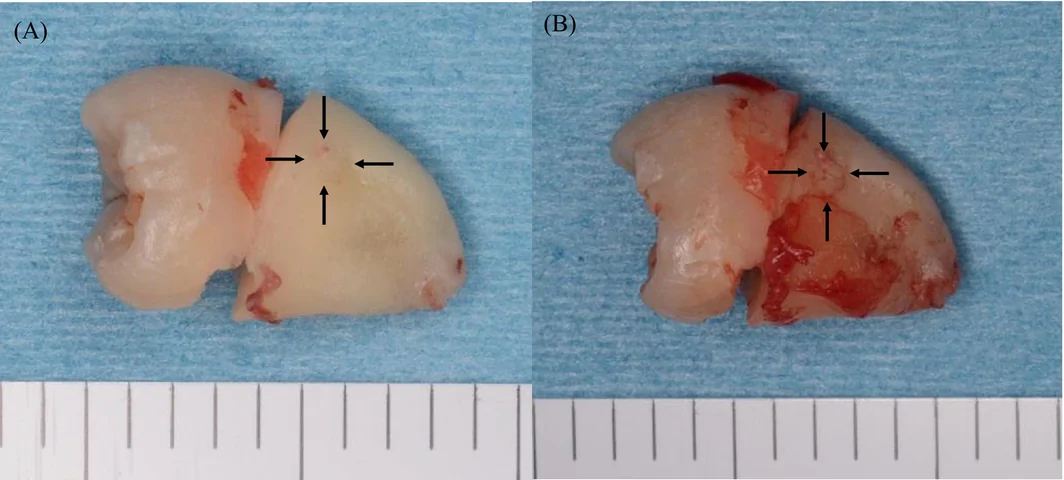
Maxillary left third molar that was subjected to split extraction. The raised structure of enamel pearl was observed at the region indicated by the arrow.

On December 2, 2024, the maxillary left third molar was extracted through specific procedures. Infiltration anesthesia was performed with 3.6 mL of ORA injection (GC Showayakuhin, Tokyo, Japan) containing lidocaine with 1:80,000 epinephrine and the mesial buccal and the distal buccal gingiva of the maxillary left second molar were incised. A mucoperiosteal flap was formed and peeled back, and the bone surrounding the maxillary left third molar was removed, followed by the split extraction of the maxillary left third molar (Figure 5). The procedure was completed with five sutures using a silk thread (NESCOSUTURE Silk Braid, Alfresa Pharma, Osaka, Japan). During the surgery, we observed a slight perforation into the maxillary sinus. The sutures were removed on December 13, 2024. After the tooth extraction, the patient experienced no sensation of water leakage from the nose when drinking water or similar symptoms when cleaning the wound.

## 4 Conclusion and Results

**FIGURE 6:**
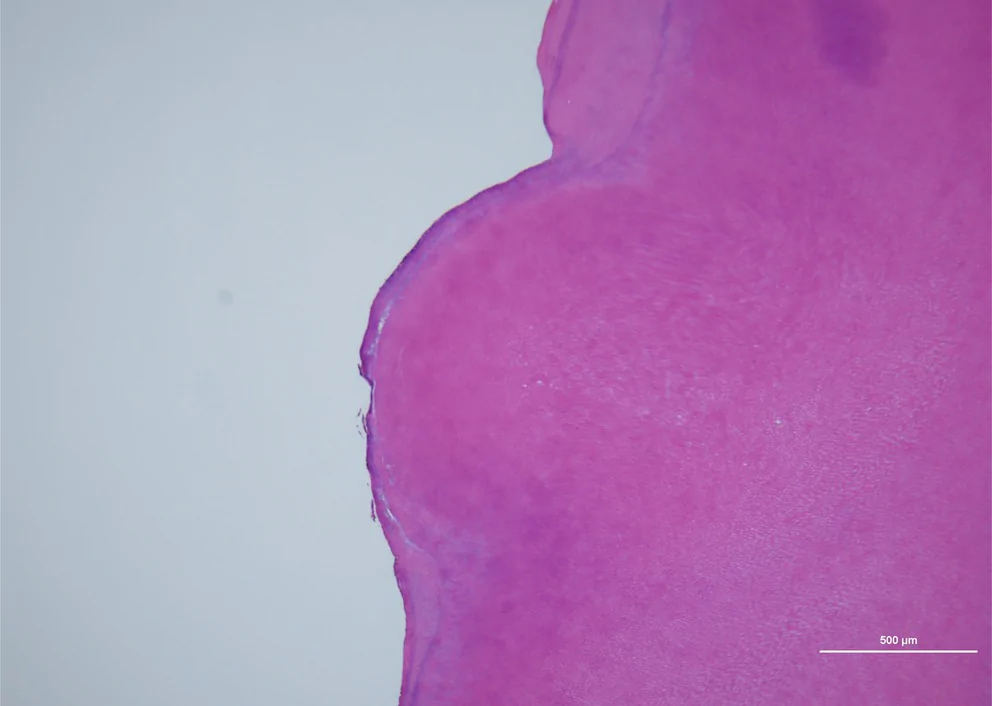
Site of the enamel pearls after demineralization. The enamel pearls were of the enamel‐dentin type.

Subsequently, we histologically examined the interior of the enamel pearls using demineralized tissue sections. The enamel pearls contained dentin, indicating that they were of the enamel‐dentin type (Figure 6).

In conclusion, enamel pearls associated with impacted maxillary third molars may elevate or partially perforate the maxillary sinus floor and may not be detectable on panoramic radiographs alone. This case illustrates the clinical value of CBCT imaging for identifying enamel pearls and accurately assessing their relationship with the maxillary sinus before extraction. Preoperative CBCT evaluation may support safer surgical planning and help prevent complications such as sinus perforation, sinus perforation area spreading, or retention of fractured enamel pearls within the maxillary sinus.

## 5 Discussion

The present case illustrates the clinical implications of enamel pearls for the surgical management of impacted maxillary third molars located close to the maxillary sinus floor. Specifically, enamel pearls may elevate or partially perforate the sinus floor, thereby increasing the risk of sinus perforation, sinus perforation area spreading, enamel pearl fracture, displacement into the sinus cavity during extraction as well as postoperative complications. These findings also emphasize the value of CBCT imaging for accurate preoperative assessment and surgical planning in such cases.

Enamel pearls are associated with plaque deposition, causing the development of periodontal disease [3, 4, 5]. Such enamel pearls require removal and treatment. However, the clinical significance of molars, which are frequently impacted and have a high incidence of enamel pearls, is largely unexplored.

A tooth extraction is performed by pushing the tooth along the root with tooth extraction forceps (elevator) using the mechanical advantage of a wedge, which induces tooth luxation [17]. However, as in the present case, if the axis of an impacted wisdom tooth is not facing the occlusal plane, its extraction requires gingival incision, tooth splitting (if necessary), and root luxation. A tooth extraction elevator is used in cases requiring luxation; however, if the enamel pearls are on the side where a tooth extraction elevator is inserted, the elevator insertion could directly cause the breakage of the enamel pearls, which may remain in the extraction socket or migrate into the maxillary sinus. Similarly, if the enamel pearls are on the side opposite to where a tooth extraction elevator is inserted, the elevator insertion applies pressure to the lateral side of the tooth root and a force toward the tooth crown, which likely causes the fracture of the enamel pearls or invasion of the alveolar bone.

Enamel pearls are 0.3–4.0 mm in size [6, 7, 8, 9]. In addition, most enamel pearls are of the enamel‐dentin type, while those with a small diameter (0.3 mm) are of the enamel type [6, 7]. The enamel pearls in the present case, which exceeded 1 mm in size, contained dentin and were of the enamel‐dentin type, an observation consistent with previous reports.

In the present case, MDCT images indicated the presence of a highly radiation‐absorbing material attached to the tooth root; however, the CT value was slightly lower than that of normal enamel. This was likely owing to the partial volume effect, as the enamel pearls were small. Furthermore, MDCT images did not sufficiently reveal the relationship among the tooth root, enamel pearls, and maxillary sinus floor. In contrast, CBCT images revealed the presence of enamel pearls, as well as their positional relationship with the maxillary sinus floor and suggested the partial rupture of the maxillary sinus floor.

This report contains several limitations. First, dental facilities and practices performing maxillary third molar extractions do not necessarily have the infrastructure in place to perform CBCT scans. Second, although identifying enamel pearls on panoramic radiographs is challenging, performing CBCT scans for all maxillary third molar extractions would increase patient radiation exposure. Nevertheless, CBCT imaging remains effective not only for confirming the presence of enamel pearls but also for clarifying the relationship between the maxillary sinus and the maxillary third molar, particularly in cases where the root contacts the sinus floor. From a clinical perspective, CBCT imaging may be particularly useful in cases in which impacted maxillary third molars are in close proximity to the maxillary sinus floor, show unusual root morphology, or require complex surgical extraction. In such situations, three‐dimensional assessment may contribute to safer surgical procedures and reduce postoperative complications.

## Author Contributions

**Hideki Suito:** conceptualization, project administration, supervision, writing – original draft, writing – review and editing. **Yuuri Oku:** conceptualization, writing – original draft, writing – review and editing. **Tomoyuki Kondo:** investigation, writing – original draft, writing – review and editing. **Kumiko Kamada:** investigation, writing – review and editing. **Naito Kurio:** investigation, visualization, writing – review and editing. **Naoki Maeda:** conceptualization, project administration, supervision, writing – original draft, writing – review and editing.

## Funding

The authors have nothing to report.

## Consent

Written informed consent was obtained from the patients to publish this report in accordance with the journal's patient consent policy.

## Conflicts of Interest

The authors declare no conflicts of interest.

## Data Availability

Data available on request from the authors The data that support the findings of this study are available from the corresponding author upon reasonable request.
